# Pharmacological removal of serum amyloid P component from intracerebral plaques and cerebrovascular Aβ amyloid deposits *in vivo*

**DOI:** 10.1098/rsob.150202

**Published:** 2016-02-03

**Authors:** Raya Al-Shawi, Glenys A. Tennent, David J. Millar, Angela Richard-Londt, Sebastian Brandner, David J. Werring, J. Paul Simons, Mark B. Pepys

**Affiliations:** 1 Wolfson Drug Discovery Unit, Centre for Amyloidosis and Acute Phase Proteins, Division of Medicine, Royal Free Campus, University College London, Rowland Hill Street, London NW3 2PF, UK; 2 Division of Neuropathology and Department of Neurodegenerative Disease, Institute of Neurology, University College London, Queen Square, London WC1N 3BG, UK; 3 Stroke Research Group, Department of Brain Repair and Rehabilitation, Institute of Neurology, University College London, Queen Square, London WC1N 3BG, UK

**Keywords:** Alzheimer's disease, Aβ amyloid, serum amyloid P component, cerebral amyloid angiopathy, CPHPC

## Abstract

Human amyloid deposits always contain the normal plasma protein serum amyloid P component (SAP), owing to its avid but reversible binding to all amyloid fibrils, including the amyloid β (Aβ) fibrils in the cerebral parenchyma plaques and cerebrovascular amyloid deposits of Alzheimer's disease (AD) and cerebral amyloid angiopathy (CAA). SAP promotes amyloid fibril formation *in vitro*, contributes to persistence of amyloid *in vivo* and is also itself directly toxic to cerebral neurons. We therefore developed (R)-1-[6-[(R)-2-carboxy-pyrrolidin-1-yl]-6-oxo-hexanoyl]pyrrolidine-2-carboxylic acid (CPHPC), a drug that removes SAP from the blood, and thereby also from the cerebrospinal fluid (CSF), in patients with AD. Here we report that, after introduction of transgenic human SAP expression in the TASTPM double transgenic mouse model of AD, all the amyloid deposits contained human SAP. Depletion of circulating human SAP by CPHPC administration in these mice removed all detectable human SAP from both the intracerebral and cerebrovascular amyloid. The demonstration that removal of SAP from the blood and CSF also removes it from these amyloid deposits crucially validates the strategy of the forthcoming ‘Depletion of serum amyloid P component in Alzheimer's disease (DESPIAD)’ clinical trial of CPHPC. The results also strongly support clinical testing of CPHPC in patients with CAA.

## 1. Background

The amyloid fibrils which are deposited as the pathognomonic intracerebral amyloid plaques and cerebrovascular amyloid deposits in Alzheimer's disease (AD) are composed of the Aβ peptide [1]. Aβ is derived by sequential proteolytic cleavage of the amyloid precursor protein (APP) first by β-secretase [2], and subsequently by the γ-secretase complex, of which the presenilin proteins (PSEN 1, PSEN 2) are the catalytic subunits [3]. Autosomal dominant hereditary AD is caused by mutations in, or duplication of, the *APP* gene and by mutations in the *PSEN 1*, *PSEN 2* genes [4]. The APP–Aβ pathway thus must have a causal role in neurodegeneration but neither the actual pathogenic moiety nor the mechanisms underlying neuronal damage in AD are known and all attempts so far at therapeutic targeting of this pathway have failed through lack of efficacy and/or drug toxicity [5]. In contrast, the Aβ amyloid in sporadic cerebral amyloid angiopathy (CAA) clearly causes the structural disruption which results in vascular dysfunction and cerebral haemorrhage, just as the amyloid deposits in the tissues cause disease in systemic amyloidosis [6]. Indeed, CAA is by the far the most common and serious form of local amyloidosis.

Serum amyloid P component (SAP) has long been identified by our laboratory as a therapeutic target in all forms of amyloid deposition [7–9]. SAP is an invariant, normal, highly proteinase resistant, plasma glycoprotein which is always present in all human amyloid deposits [10], including the Aβ amyloid of AD and CAA [11–13]. SAP binds avidly but reversibly to all amyloid fibrils, including those which are the direct cause of disease in systemic amyloidosis [6] and in CAA [14], and those in AD where their role in pathogenesis, if any, is not known. Binding of SAP promotes amyloid fibrillogenesis *in vitro* [15,16] and stabilizes the fibrils, protecting them from proteolytic cleavage and acting as an anti-opsonin to prevent their destruction by phagocytic cells [8,17]. SAP thus is likely to contribute significantly to the unique persistence of amyloid deposits *in vivo* [18] that contrasts with the generally very efficient clearance of autologous debris from the extracellular space.

In addition, unrelated to its role in amyloid deposits, human SAP is directly neurotoxic to cerebral neurons [19–21]. It binds to them, is internalized, traffics to the nucleus [22] where it binds to chromatin [23], and leads to apoptotic cell death. SAP is synthesized and catabolized only by the liver [24] and human SAP is not expressed in the brain [25], which it enters only from the blood. It is normally present in the cerebrospinal fluid (CSF) at only about one-thousandth of the plasma concentration [26,27]. The binding of SAP to intracerebral Aβ amyloid deposits and plaques must increase the brain content of SAP [28]. The reversible non-covalent interaction with amyloid fibrils [10] must then also provide an increased local concentration of free SAP adjacent to amyloid deposits. Furthermore, other known risk factors for dementia, including advanced age, cerebral haemorrhage, traumatic brain injury (TBI) and severe or repeated non-penetrating head injury, all, certainly or probably, increase exposure of the brain to SAP, which could then contribute to neurodegeneration. In old age, the increase is simply the prolonged duration of exposure to normal CSF SAP concentrations but the other conditions, with compromised cerebral vascular integrity, enable plasma, with its 1000-fold higher SAP concentration, to enter the cerebral substance.

Our drug, (R)-1-[6-[(R)-2-carboxy-pyrrolidin-1-yl]-6-oxo-hexanoyl]pyrrolidine-2-carboxylic acid (CPHPC), was developed to abrogate the pathogenic contribution of human SAP to amyloid formation and persistence [9]. CPHPC is specifically bound by human SAP in the circulation to form stable complexes of pairs of native pentameric SAP molecules cross-linked by the drug [9]. These complexes are immediately cleared by the liver, leading safely and effectively to almost complete depletion of plasma SAP for as long as the drug is administered [9,29]. We have shown that depletion of plasma SAP by administration of CPHPC to patients with AD leads to complete disappearance of SAP from the CSF [30]. CPHPC also enters the CSF in AD patients [30] and is thus available to inhibit binding of SAP to amyloid fibrils. Hence the rationale for University College London's forthcoming ‘Depletion of serum amyloid P component in Alzheimer's disease (DESPIAD)’ clinical trial of CPHPC in AD is: (i) that removal of all SAP from the brain by CPHPC will abrogate the direct neurotoxicity of human SAP; and (ii) that stripping of SAP from the cerebral plaques and cerebrovascular Aβ amyloid deposits will promote amyloid clearance. The same rationale underpins further planned clinical studies of CPHPC in CAA and TBI.

We already know that the first goal of removing all SAP from the CSF will be achieved [30], but it is obviously not possible to directly demonstrate removal of SAP from amyloid deposits in living human subjects. Indeed, although CPHPC treatment very substantially reduces the amount of SAP in systemic amyloid deposits, the affinity of binding of SAP to CPHPC is not sufficient for the drug to strip all SAP from its avid multivalent binding to the very abundant amyloid deposits that are present in these patients [29]. In contrast, cerebral and cerebrovascular amyloid deposits are several orders of magnitude less abundant than the deposits in systemic amyloidosis. Also the intracerebral plaques are exposed to a 1000-fold lower SAP concentration than is present in the plasma. On purely thermodynamic grounds, therefore, CPHPC should completely remove SAP from cerebral Aβ amyloid plaques and CAA deposits in patients.

Mouse SAP binds about 30-fold more weakly than human SAP to all known ligands so that, although mouse SAP is present in murine systemic amyloid deposits, it is not found in cerebral Aβ amyloid in mouse models of human AD [31], and circulating mouse SAP is not depleted by CPHPC *in vivo* [9]. In order to investigate the capacity of CPHPC to remove bound SAP from amyloid deposits in the brain, we therefore introduced transgenic expression of human SAP into the TASTPM double transgenic mouse model of familial AD [32]. The TAS and TPM transgenes encode pathogenic variants of human APP and human presenilin-1, respectively, and the mice develop abundant intracerebral and cerebrovascular human Aβ amyloid deposits. Here we report that the triple transgenic mice showed the expected presence of human SAP in all their amyloid deposits and that it was completely removed by sustained depletion of circulating human SAP produced by long-term administration of CPHPC.

## 2. Material and methods

### 2.1. Human CAA tissue

Formalin fixed wax embedded blocks of cerebral cortex tissues from nine patients with histologically confirmed sporadic CAA were provided by the Queen Square Brain Bank for Neurological Disorders, UCL.

### 2.2. Transgenic mice

TASTPM transgenic mice [32], homozygous for both the TAS and TPM transgenes, were provided by GlaxoSmithKline. The human SAP gene was amplified by PCR using Phusion High-Fidelity DNA Polymerase (NEB) and cloned in a plasmid vector as a 2.6 kb *Hin*dIII fragment, comprising the 1 kb SAP gene with 0.6 and 1 kb of 5′ and 3′ flanking sequence, respectively. After verifying the exon sequences, the 2.6 kb *Hin*dIII insert was released from the vector, gel purified and microinjected into pronuclei of C57BL/6J mouse embryos to generate transgenic mice expressing human SAP on the inbred C57BL/6J background. Lines were established and maintained hemizygous by backcrossing to wild-type C57BL/6J partners. TASTPM, human SAP triple transgenic mice were generated by crossing TASTPM homozygous mice with human SAP transgenic mice. The progeny were thus either hemizygous for all three transgenes or were hemizygous for the TAS and TPM transgenes. Genotypes were assessed by PCR. Data shown are representative of findings with both males and females.

### 2.3. Immunoassay for SAP

SAP protein concentrations were assayed by electroimmunoassay or immunoradiometric assay, as described previously [27,33].

### 2.4. Immunohistochemical staining for Aβ and SAP

Immunoperoxidase histochemistry was performed on a Ventana Discovery XT staining platform using the Ventana DAB Map Kit. Wax sections of formalin fixed tissue were pretreated with formic acid (for Aβ staining) or with Ventana Protease 3 (for SAP staining) and blocked for 8 min using Superblock (Medite). A mouse monoclonal primary antibody was used for detection of Aβ (Dako, clone 6F-3D), and an in-house monospecific polyclonal rabbit antiserum was used to detect human SAP, and counterstained with haematoxylin. Immunofluorescence histochemistry was performed on 14 µm frozen sections of unfixed frozen mouse brains. Sections were dried at room temperature and stored at −20°C before use. Non-specific binding was blocked with 2% w/v BSA, 0.1% Triton X-100 in PBS, and SAP was then detected with the monospecific rabbit anti-human SAP antibody followed by Alexa-488 labelled anti-rabbit IgG antibody, followed by counterstaining with bisbenzimide.

### 2.5. Histochemical staining for amyloid

Amyloid deposits in histological sections were stained with Congo red [34] and identified by their pathognomonic green birefringence in high intensity cross polarized light.

## 3. Results

### 3.1. SAP in human cerebral and cerebrovascular Aβ amyloid deposits

**Figure 1.**
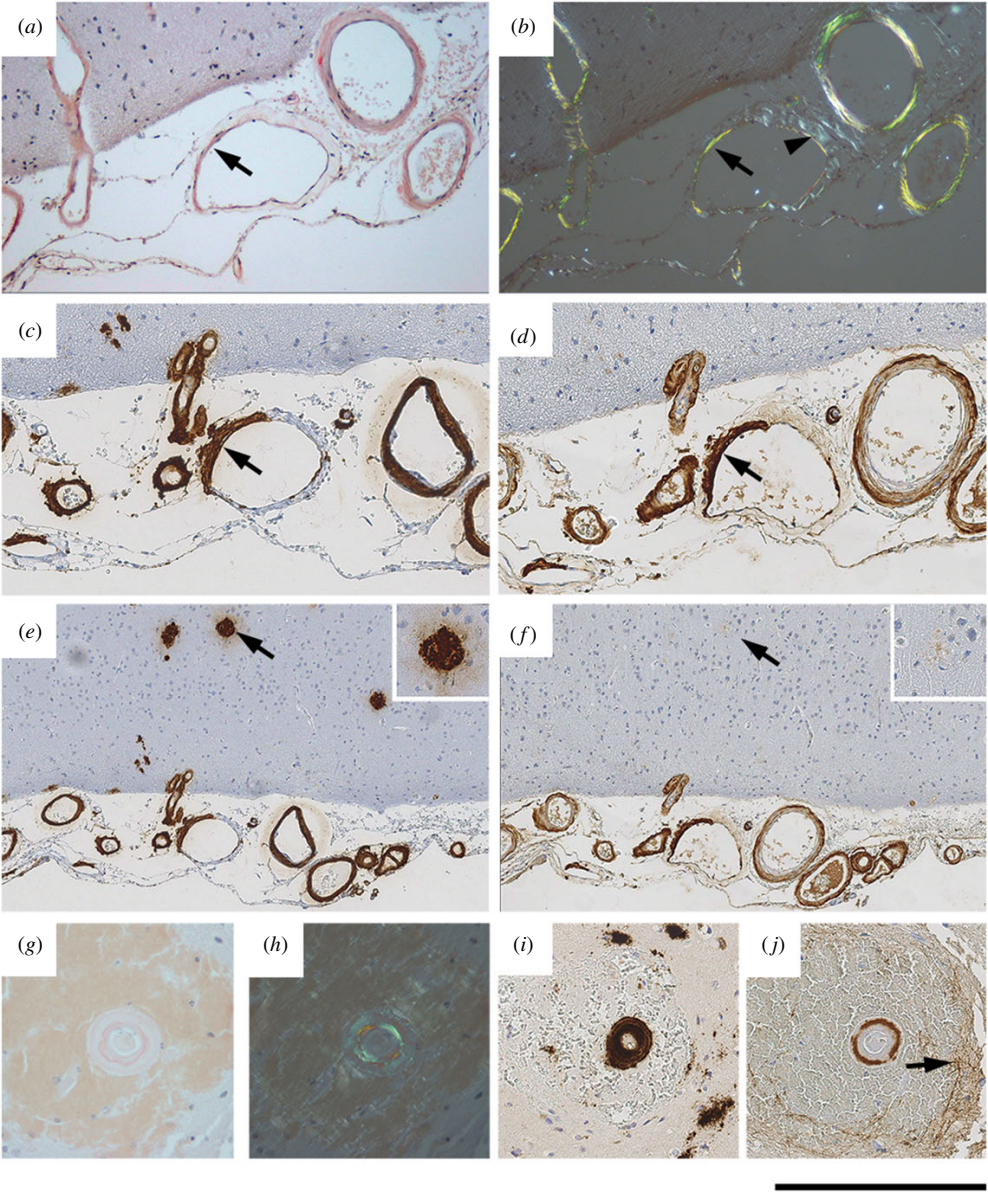
Localization of SAP in human CAA. (*a*,*b*) Congo red stained section viewed with bright field illumination (*a*) and with intense crossed polarized light (*b*), showing abundant amyloid in blood vessel walls. The arrowhead in (*b*) shows the white birefringence of collagen often seen in normal blood vessels, in contrast to the green birefringence of Congo red stained amyloid. (*c*,*d*) Immunoperoxidase staining for Aβ (*c*) and SAP (*d*), showing strong immunoreactivity in vessel walls. In one vessel, the amyloid is mostly confined to one segment of the vessel wall (arrow in *a*–*d*). (*e*,*f*) Lower magnification images of the sections shown in (*c*,*d*), showing amyloid plaques which stain strongly for Aβ and very weakly for SAP. Higher magnification images of the arrowed plaque are inset. (*g*–*j*) Sections of an amyloid-containing blood vessel surrounded by blood. (*g*,*h*) Congo red stained section viewed with bright field illumination (*g*) and with intense cross polarized light (*h*). (*i*,*j*) Immunostaining for Aβ and SAP, respectively. The arrow in (*j*) shows diffuse staining in adjacent neural tissue, not associated with amyloid, likely due to a microbleed. Scale bars, (*a*–*d*), 250 µm; (*e*,*f*), 500 µm; (*g*–*j*), 250 µm.

The universal presence of human SAP in Aβ amyloid has been extensively confirmed since our original 1984 observations on sporadic and hereditary CAA [11]. However, in order to validate our methods for subsequent application to studies in human SAP transgenic mice, we examined brain sections from nine confirmed sporadic CAA patients using Congo red staining for amyloid and immunohistochemical staining of adjacent sections for SAP and Aβ. In each human case, SAP and Aβ staining were observed in congruent patterns in blood vessel walls (figure 1), and in eight of the nine cases, the SAP staining was strong. In seven of the cases, intracerebral Aβ amyloid plaques were also present. They stained with anti-SAP antibodies but much less intensely than the vascular amyloid in the same sections (figure 1*e,f*). Diffuse immunostaining for SAP was also seen in perivascular areas (figure 1*g*–*j*), presumably reflecting recent vascular leakage.

### 3.2. Aβ amyloid deposition in TASTPM mice

**Figure 2.**
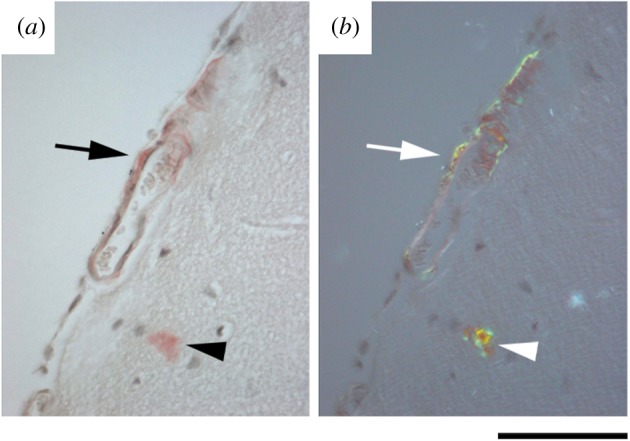
Cerebrovascular amyloid in TASTPM mice. Unfixed frozen brain sections from six-month-old female TASTPM mouse stained with Congo red showing cerebrovascular amyloid (arrow) and cerebral plaque (arrowhead). (*a*) Bright field illumination; (*b*) intense cross polarized light showing pathognomonic green birefringence of Congo red bound to amyloid. Scale bar, 150 µm.

Almost all patients with AD have CAA, although usually of lesser severity than in patients with typical sporadic CAA and predominantly affecting capillaries rather than arterioles. Similar cerebrovascular amyloid deposition is seen in mouse models of AD [31,35]. The double transgenic TASTPM mice have been reported to develop immunologically detectable human Aβ deposits from three months of age and amyloid plaques by six months [32]. Here we also observed extensive vascular amyloid deposition at six months of age in TASTPM mouse brains, with pathognomonic green birefringence in polarized light of both plaques and cerebral blood vessels stained with Congo red (figure 2).

### 3.3. Human SAP transgenic mice and CPHPC administration

**Figure 3.**
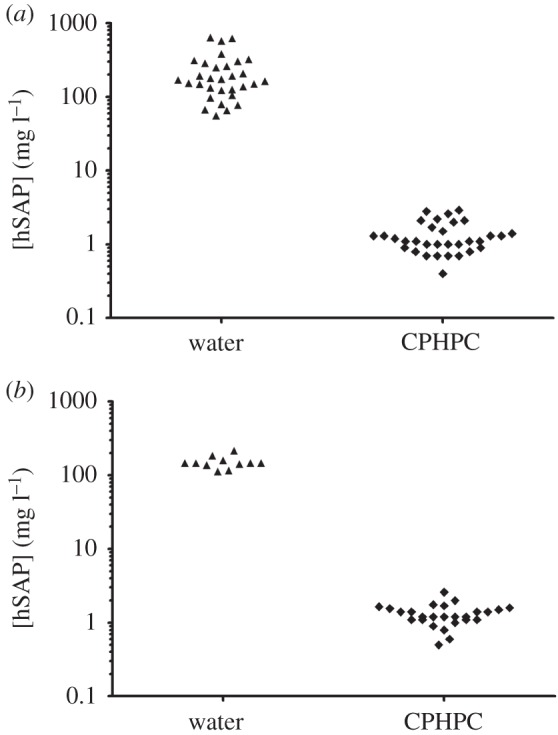
Human SAP expression in transgenic mice, and depletion by CPHPC. Human SAP concentrations in sera of individual transgenic mice, while on water alone or water containing 5 mg ml^−1^ CPHPC. (*a*) Line 38 human SAP transgenic C57BL/6 mice; (*b*) line 38 human SAP transgenic TASTPM mice. Results are from males and females with no apparent differences between sexes.

Several human SAP transgenic lines were established, two of which were expanded for further study. The serum concentration of human SAP in line 9 transgenic mice was mean (s.d.) 29.2 (4.0) mg l^−1^ (*n* = 6), closely corresponding to the human reference range [27]. In contrast, the human SAP concentration in sera of line 38 transgenic mice was mean (s.d.) 216.9 (154.9) mg l^−1^ (*n* = 31) (figure 3*a*), with no statistically significant difference between males and females. Following administration to line 38 mice of CPHPC at 5 mg ml^−1^ in their drinking water, the serum concentration of human SAP rapidly fell to 1.33 (0.64) mg l^−1^ (*n* = 32) (figure 3*a*) and remained reduced by more than 97% for as long as drug exposure continued.

### 3.4. Human SAP transgenic TASTPM mice

**Figure 4.**
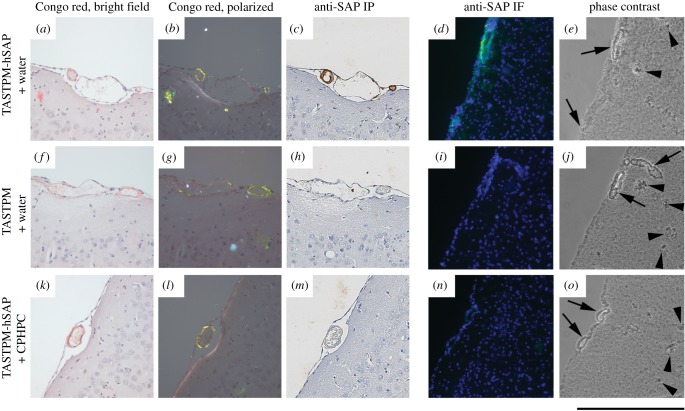
Depletion of human SAP from cerebrovascular amyloid by CPHPC treatment of transgenic mice. Sections of formalin fixed wax embedded cerebral cortex from 20-month-old mice stained with Congo red and viewed in bright field (*a*,*f*,*k*) or intense cross polarized illumination (*b*,*g*,*l*), and also immunoperoxidase (IP) stained with anti-human SAP antibodies (*c*,*h*,*m*). Unfixed frozen sections from the same tissues stained by immunofluorescence (IF) with anti-human SAP antibodies (green) and bisbenzimide counterstain (blue) (*d*,*i*,*n*) and also viewed in phase contrast (*e*,*j*,*o*). (*a*–*e*) Human SAP transgenic TASTPM mouse, (*f*–*j*) TASTPM control mouse, (*k*–*o*) CPHPC-treated human SAP transgenic TASTPM mouse. Amyloid-containing blood vessels are labelled with arrows, and example amyloid plaques are indicated by arrowheads. Tissues in (*a*–*c*), (*f*–*h*), (*k*–*m*) are from males and (*d*,*e*,*i*,*j*,*n*,*o*) from female mice.

After introduction of the human SAP transgene into TASTPM mice by crossing them with line 38, the circulating concentration of human SAP was in the same range as in the transgenic mice on the wild-type background (figure 3*b*). When examined at 20 months of age, both the vascular and plaque Aβ amyloid deposits were positive for human SAP by immunoperoxidase staining of sections of formalin fixed wax embedded brain (figure 4*a*–*c*), while control sections from age matched TASTPM mice without the human SAP transgene were entirely negative (figure 4*f*–*h*). This was confirmed by immunofluorescence staining of frozen sections of unfixed tissue from human SAP transgenic TASTPM mice (figure 4*d*,*e*) and control TASTPM animals (figure 4*i*,*j*).

### 3.5. Effects of CPHPC administration and SAP depletion

Administration to human SAP transgenic TASTPM mice of 5 mg ml^−1^ of CPHPC in drinking water, from age four or six months until age 20 months, had no discernible adverse effects. The body weights of CPHPC-treated mice were indistinguishable from those of controls (data not shown). The circulating human SAP concentration in CPHPC-treated animals fell to between 0.1 and 2.9 mg l^−1^ in samples taken during treatment and at termination (figure 3*b*). Furthermore, no immunoperoxidase staining for human SAP was observed in the amyloid deposits after CPHPC treatment (figure 4*k*–*m*), in marked contrast to the positive staining in untreated human SAP transgenic TASTPM mice (figure 4*a*–*c*). The immunoreactivity of human SAP is diminished by standard formalin fixation and wax embedding [36], and antigen retrieval techniques are required for its optimal detection by immunoperoxidase staining. The presence of human SAP was therefore also sought by immunofluorescence staining of cryosections of unfixed tissue. The results confirmed the absence of appreciable staining for human SAP (figure 4*n*,*o*) with appearances indistinguishable from those of tissues from control TASTPM mice without the human SAP transgene (figure 4*i*,*j*).

### 3.6. Human SAP content of intracerebral plaques and cerebrovascular amyloid deposits

**Figure 5.**
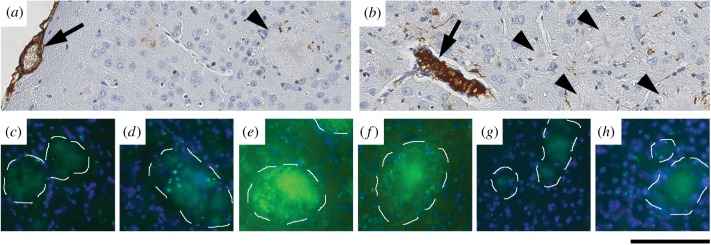
Depletion of human SAP from cerebral plaques by CPHPC treatment of transgenic mice. (*a*,*b*) Sections of formalin fixed wax embedded cerebral cortex from 20-month-old human SAP transgenic male TASTPM mice, immunoperoxidase stained with anti-human SAP antibodies, showing intense staining of vascular amyloid (arrows) and very weak staining of intracerebral plaques (arrowheads). (*c*–*h*) Amyloid plaques (outlined) in unfixed frozen sections of cerebral cortex from 20-month-old female mice stained with fluorescent labelled anti-human SAP antibodies (green). (*c*,*d*) TASTPM control; (*e*,*f*) human SAP transgenic TASTPM; (*g*,*h*) CPHPC-treated human SAP transgenic TASTPM. The green channel exposures used for panels (*c*–*h*) were 10 times longer than for figure 4*d*,*i*,*n*. At the exposure that demonstrated SAP in the cerebrovascular amyloid in figure 4, there was no signal from the intracerebral plaques. Scale bars, (*a*,*b*), 100 µm; (*c*–*h*), 150 µm.

In brain sections from human SAP transgenic TASTPM mice, anti-SAP antibodies consistently stained the cerebrovascular amyloid deposits much more intensely than the parenchymal amyloid plaques (figure 5), in both immunoperoxidase staining of formalin fixed wax embedded tissue (figure 5*a*,*b*) and immunofluorescence staining of cryosections of unfixed tissue (figure 5*e*,*f*). The SAP content of cerebrovascular amyloid is thus considerably greater than that of plaque amyloid, consistent with *in vivo* exposure of the plaques only to the SAP concentration of CSF fluid which, in humans and presumably also in mice, is about 1000-fold lower than the plasma and extracerebral interstitial fluid concentration. Using up to 10-fold longer exposures, even trace immunofluorescent staining with anti-SAP antibody could be sensitively detected (figure 5*e*,*f*) and the abundance of SAP in the deposits semi-quantitatively assessed. This confirmed that after CPHPC treatment both the cerebrovascular deposits and the cerebral plaques had been stripped of SAP (figure 5*g*,*h*), with staining intensity not different from the background autofluorescence of plaques in TASTPM controls with no human SAP transgene (figure 5*c*,*d*).

## 4. Discussion

We have previously identified human SAP as a therapeutic target in both systemic amyloidosis and in neurodegenerative conditions associated with cerebral and cerebrovascular Aβ amyloid deposition [7–9]. We have also shown that administration of the SAP depleting drug, CPHPC, produces sustained depletion of plasma SAP for as long as the drug is administered [29] and that depletion of circulating SAP leads to complete disappearance of SAP from the CSF in patients with AD [30]. Since SAP is not expressed in the human brain [25], the SAP depletion effected by CPHPC should abrogate the direct neurotoxicity of this plasma protein. However, for depletion of free soluble SAP to promote the desired enhanced clearance of amyloid deposits, it should ideally lead to essentially complete removal of SAP from its binding to amyloid fibrils. This does not happen in systemic amyloidosis because, despite the ability to safely administer high doses of this non-toxic, non-metabolized drug, the CPHPC concentrations which are attainable *in vivo* are not sufficient to reverse the avid multivalent binding of the pentameric SAP molecule to the very abundant amyloid fibrils. Even after months of daily dosing with CPHPC, some SAP, albeit a much reduced amount, remains associated with visceral amyloid deposits [29]. Although no regression of amyloid then ensues, observations in CPHPC-treated systemic amyloidosis patients suggest that the treatment nonetheless may reduce new amyloid accumulation and stabilize the function of amyloidotic organs [29].

The situation regarding cerebral and cerebrovascular Aβ amyloid is radically different. The total mass of Aβ amyloid is extremely small in comparison with systemic amyloid deposits. We therefore expected that plasma SAP depletion by CPHPC would successfully remove all SAP bound to cerebral parenchymal and vascular Aβ amyloid deposits, and this has been confirmed by the present observations. Furthermore, parenchymal plaques are exposed to about 1000-fold lower ambient SAP concentrations in the CSF [26,30] than the cerebrovascular deposits bathed in plasma-derived extracellular fluid. The relative quantities of bound SAP differ accordingly, as we have confirmed here immunohistochemically, and this explains why, over 30 years ago, we [11] and others [37] did not initially detect SAP on the cerebral parenchymal plaques using much less sensitive immunostaining procedures.

It is important to note that the present experiments were not designed to show an effect of CPHPC treatment and human SAP depletion on Aβ amyloid load. TASTPM mice strongly overexpress two different familial AD genes under the mouse Thy-1 gene promoter [32] and develop extensive cerebral amyloid plaques and CAA in the absence of human SAP expression. It is therefore unlikely that their Aβ amyloid deposition will be detectably modulated by the presence or depletion of human SAP.

The pathogenic role, if any, of the cerebral parenchymal Aβ amyloid plaques in AD and that appear soon after TBI [38] is unknown, but an intervention which leads to clearance and/or prevention of the deposits would be very informative. This is one of the aims of our existing DESPIAD clinical trial of CPHPC in patients with AD, scheduled to start in 2016. In contrast to AD, the Aβ amyloid deposits in the walls of cerebral blood vessels in CAA are unequivocally the cause of vascular dysfunction and cerebral haemorrhage in this very important and prevalent disease [14]. Progressive deposition of amyloid disrupts blood vessel structure with loss of smooth muscle cells, thickened vessel walls and eventually detachment and delamination of the outer part of the tunica media. CAA is present at autopsy in 20–40% of non-demented and 50–60% of demented elderly populations, and in at least 90% of individuals with Alzheimer's disease (AD) [14]. CAA causes microbleeds and lobar intracerebral haemorrhages, comprising up to 20% of haemorrhagic strokes [14]. CAA is also associated with ischaemic brain injury, including small areas of infarction [39] and white matter abnormalities [40], and it impairs normal vascular function [41,42]. The very high, approximately 10%, annual rate of recurrent intracerebral haemorrhage in CAA increases the long-term risk of dementia [43]. The spectrum of vascular brain injury and dysfunction in CAA is also likely to cause cognitive impairment, independently of cerebral haemorrhage [44,45]. CAA is thus a particularly attractive indication both for an intervention designed to promote clearance of amyloid and for abrogation of the direct neurotoxicity of blood-derived SAP entering the brain.
